# Does Patriline Composition Change over a Honey Bee Queen’s Lifetime?

**DOI:** 10.3390/insects3030857

**Published:** 2012-09-13

**Authors:** Robert Brodschneider, Gérard Arnold, Norbert Hrassnigg, Karl Crailsheim

**Affiliations:** 1 Karl-Franzens-University Graz, Universitätsplatz 2, A-8010 Graz, Austria; E-Mails: norbert.hrassnigg@uni-graz.at (N.H.); karl.crailsheim@uni-graz.at (K.C.); 2 CNRS, Laboratoire Evolution, Génomes et Spéciation, UPR 9034, CNRS, 91198, Gif-sur-Yvette cedex, France and Université Paris-Sud 11, 91405 Orsay cedex, France; E-Mail: gerard.arnold@legs.cnrs-gif.fr

**Keywords:** *Apis mellifera*, patriline, polyandry, spermatheca, sperm mixing, sperm competition, sperm precedence, sperm usage

## Abstract

A honey bee queen mates with a number of drones a few days after she emerges as an adult. Spermatozoa of different drones are stored in her spermatheca and used for the rest of the queen’s life to fertilize eggs. Sperm usage is thought to be random, so that the patriline distribution within a honey bee colony would remain constant over time. In this study we assigned the progeny of a naturally mated honey bee queen to patrilines using microsatellite markers at the queen’s age of two, three and four years. No significant changes in patriline distribution occurred within each of two foraging seasons, with samples taken one and five months apart, respectively. Overall and pair-wise comparisons between the three analyzed years reached significant levels. Over the three-year period we found a trend for patrilines to become more equally represented with time. It is important to note that this study was performed with a single queen, and thus individual and population variation in sperm usage patterns must be assessed. We discuss long-term changes in patriline composition due to mixing processes in the queen’s spermatheca, following incomplete mixing of different drones’ sperm after mating.

## 1. Introduction

Multiple mating by females with different males (polyandry) is widespread among insects. In eusocial insects such as honey bees and ants, for example, polyandry is an especially interesting phenomenon: polyandry reduces the average intracolonial worker relationship resulting from their haplo-diploid genetic system [1,2]. Eusociality in hymenopterans is thought to have evolved under monandrous conditions and multiple matings occur after workers lose their reproductive totipotency [3]. Many hypotheses have been proposed to explain the potential benefits of polyandry, among them the genetic diversity hypothesis [4,5,6]. This hypothesis proposes that multiple mating is adaptive because of benefits gained through the increased genotypic variation within a colony. This was shown for several parameters: polyandry reduces the parasite load in honey bees [7] and bumblebees [8] and increases colony performance [9,10,11]. Polyandry also expands the range of environmental conditions tolerated by a honey bee colony through genetic variation within colonies [4,12]. Additionally, multiple mating is thought to have evolved because it reduces the risk of producing nonviable diploid males in a colony [13,14]. 

Honey bees are known to be extremely polyandrous. The estimated number of matings in the genus *Apis* ranges from 5 to more than 20, depending on the species and according to different authors and methods of investigation [15,16,17,18,19,20,21,22]. A honey bee colony typically has a single long-lived queen which produces about 150,000 progeny per year, mainly female workers, and 5,000 to 20,000 drones, which are the colony’s male offspring [23].

All workers in a colony that have not joined it through drifting [24] share the same mother but may have different fathers because of polyandry. Female offspring sharing the same father belong to the same patriline (or subfamily). Daughters of each drone are super-sisters having, on average, 75% of their genes in common by descent, whereas daughters of different fathers are half-sisters and have, on average, only 25% of their genes in common by descent [25]. The exact average genetic relationship of workers within a colony is affected by the number of matings, the distribution of sperm in the queen’s storage organ (spermatheca) and the utilization of sperm by the queen [1].

Honey bee queens mate in the course of one or more nuptial flights [26] shortly after they emerge as adults. A virgin queen rapidly mates with a variable number of drones and receives semen into the median and distensible lateral oviducts, where it is stored until her return to the colony. After her return to the hive only a small portion of the spermatozoa migrate into the spermatheca by active and passive mechanisms over a period of about 40 h [27], while up to 95% of the received semen is expelled [28]. Approximately 5 to 7 million spermatozoa [29,30] are stored in the spermatheca for the rest of the queen’s life.

Taber [15] concluded from his experiments that spermatozoa clump or stick together, and thus sperm of different drones does not mix appreciably. This would lead to successive usage of the sperm of drones and consequently would increase the level of genetic relationship of contemporary nestmates in the colony, and would in this way “restore” the conditions favorable to kin selection. Kerr *et al*. [31] report genetic fluctuations in honey bee colonies, determined by using two morphologically distinct worker genotypes, indicating that changes in patriline composition or proportions are also due to processes in the queen’s spermatheca.

Estoup *et al*. [18] established the use of hypervariable genetic markers to characterize all the patrilines of a honey bee colony. Several studies have used this tool to investigate the consequences of polyandry for sperm usage (the sequence in which sperm from different males is used for fertilization, [32]) and kin recognition [33,34], or to find evidence for genotypic task specialisation [35,36,37,38]. All these studies clearly demonstrate that many patrilines are present simultaneously in a honey bee colony, and therefore the scenario of “total” sperm clumping, *i.e.*, the usage of most of one drone’s spermatozoa before using the spermatozoa of other drones, is rightly considered to be wrong.

All authors report unequal patriline distribution, a drone’s offspring ranging from less than 1% to 32% (e.g., [22,39]) of a colony’s population. Recent studies, using microsatellite techniques, failed to find evidence of any form of sperm clumping or non-random sperm usage: Haberl and Tautz [32] point out that no sperm clumping can be detected on a fine scale. They found that sequentially laid eggs were not fertilized by sperm of the same father with a higher probability than predicted by the ratios of patrilines. A higher probability would have resulted in a higher number of same subfamily pairs of eggs. On a somewhat larger time scale, Franck *et al*. [39] studied the first three months of offspring production by an artificially inseminated queen: samples were taken three times and only samples taken more than two months apart give significant differences in patriline representation. Schlüns *et al*. [40] report unequal sperm usage one month after instrumental insemination and dependence of patriline frequency on inseminated semen volume.

Previously, no study has used microsatellite analysis to determine whether a colony is composed of patrilines in similar proportions over a long period of time or whether the proportions change. Changes in patriline composition could be the result of (a) adaptions to sperm competition [41], (b) sperm choice by the queen, (c) sperm clumping as described by Taber [42] or (d) slow mixing processes in the spermatheca, so that sperm of different drones are not completely mixed at the beginning of oviposition. If sperm are used randomly, with none of these mechanisms involved, the composition of patrilines should remain constant for the queen’s lifetime.

In our study we analyzed the patrilines of a honey bee colony by means of microsatellite markers over a period of 3 years, starting with a two-year-old queen. We addressed the questions of whether there are any variations in distribution of patrilines (i) within a foraging season and (ii) between years.

## 2. Experimental Section

### 2.1. Origin of Bees

The experiment was conducted between September 1998 and July 2000 using a honey bee colony (*Apis mellifera carnica*) with a naturally mated queen obtained from the Institut für Bienenkunde in Lunz/See. The queen started laying eggs on the 10th of August 1997, indicating that mating occurred most probably a few days before that date. The queen’s progeny were sampled on September 17th1998 (n = 165), April 20th 1999 (n = 428), September 17th 1999 (n = 128), and June 6th 2000 (n = 213) and July 5th 2000 (n = 48). Workers were taken randomly at night from different locations in the hive, to represent the overall subfamily composition of the colony. Worker samples were stored in 96% alcohol until extraction of DNA.

### 2.2. DNA Amplification and Genetic Analysis

DNA was extracted from the heads of individual worker bees. Tissues were homogenized using liquid nitrogen. Two types of DNA extraction were performed: using phenol, according to Garnery *et al*. [43] or using chelex, according to Franck *et al*. [39]. The tissues were digested after addition of 18 µL proteinase K for 1 h at 55 °C.

Four highly variable microsatellite loci (A29, A76, A107, B124) were used to assign the workers to their respective subfamilies. Polymerase chain reactions were carried out in a 10 µL reaction mixture using incorporation of ^33^P-labelled dATP [18]. The loci were amplified using a Biometra Tgradient thermocycler through 30 cycles, consisting of denaturation for 30 s at 94 °C, annealing for 30 s at 54–58 °C (depending on the locus), and elongation for 30 s at 72 °C. A sample of 2 µL of each reaction was run on 6% polyacrylamide sequence gels. The gels were dried and exposed to film according to the radiation for 10 to 48 h.

### 2.3. Statistical Analysis

All comparisons of patriline distribution were tested via R × C Fisher’s exact test (Monte-Carlo simulation, 15,000 runs, in P-Stat [44]. To test for equal representation of patrilines Chi^2^ tests were performed. 

## 3. Results

In the experimental colony, 17 patrilines were detected. Patrilines were named according to their frequency in the first year. No offspring of patriline “P”, which accounted for 1.21% of the workers in 1999, were detected in the following years. For males with very low paternity contributions, the non-sampling probability is significant: low numbers of samples can reduce the accuracy of results and can therefore be the cause for rare patrilines to be overlooked.

To calculate the non-sampling error we used (1 − *p*)*^n^* ≤ 0.05, with *p* giving the portion of a subfamily and *n* the sample size [45]. In a sample of 165 bees (which was the sample size for the first analyzed year) at least one worker of a subfamily exceeding 1.8% was sampled allowing for a non-sampling error of ≤0.05. In the second and third years the higher sampling numbers meant that smaller patriline portions (0.53% and 1.1%, respectively) could be detected with the same non-sampling probability of 5%.

**Figure 1:**
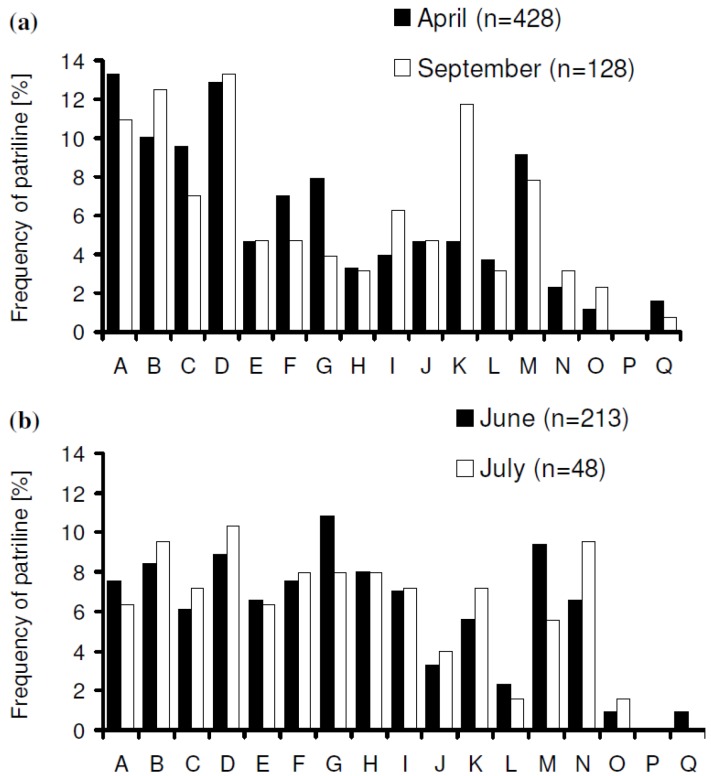
Patriline proportions in two foraging seasons. (**a**) April–September 1999, *p* > 0.5 (Fisher’s exact test) (**b**) June–July 2000, *p *> 0.35 (Fisher’s exact test).

**Figure 2:**
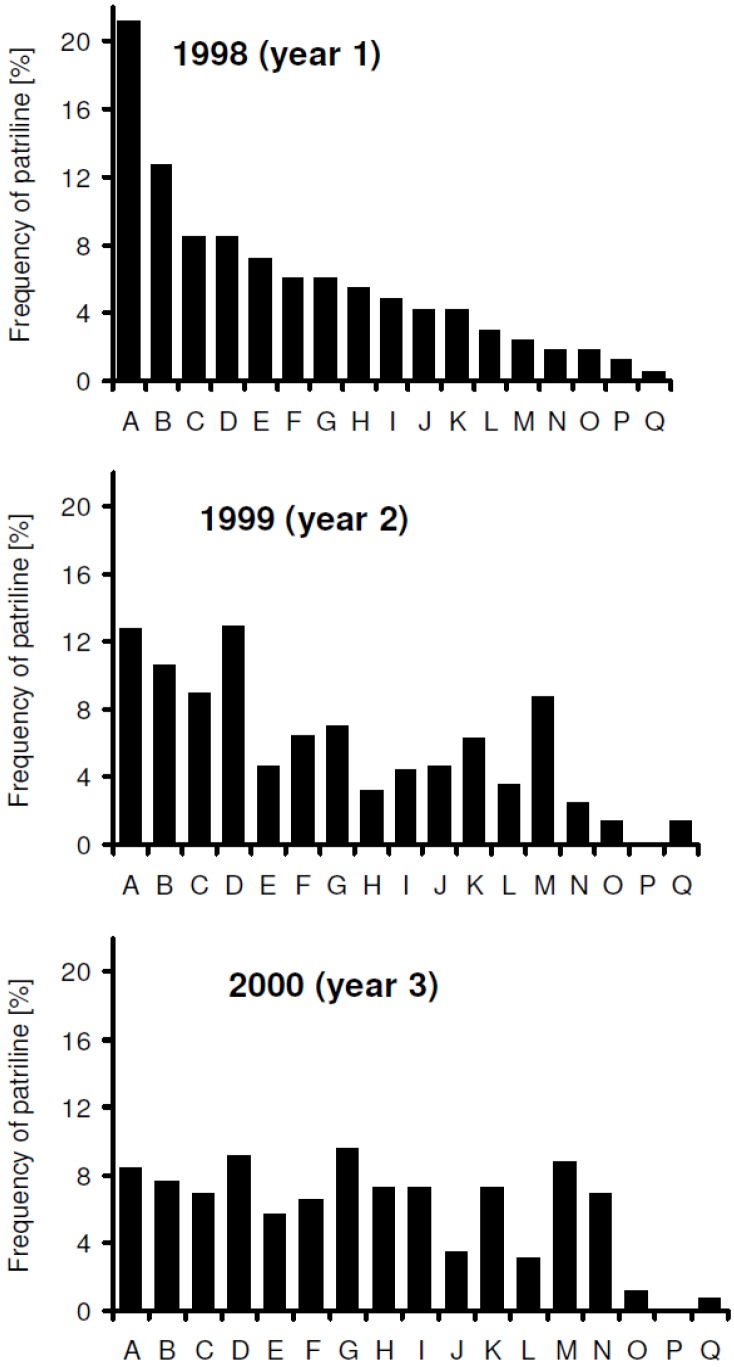
Relative frequencies of workers belonging to each patriline among the offspring of a single queen in the three years analyzed. Patrilines were named and ordered (A through Q) based on their proportions in the first year. Sample numbers were 165, 556 and 261 for the first, second and third years, respectively. Patriline distributions differed significantly between each pair of years (*p*<0.05, Fisher’s exact test).

Within one foraging season the patriline distribution did not show significant differences (April *vs*. September 1999, *p* > 0.5; and June *vs*. July 2000, *p* > 0.35; Fisher’s exact test; Figure 1a,b), whereas the overall comparison of the three analyzed years gave significant differences (*p* < 0.001, Fisher’s exact test). Also each pair-wise comparison revealed significant differences (year 1 *vs*. 2, 2 *vs*. 3, 1 *vs*. 3, *p* < 0.05, Fisher’s exact test; Figure 2). Every year another subfamily emerged as the most frequent (“A” in 1998, “D” in 1999 and “G” in 2000) and it has to be stressed that the respective portion of the most frequent patriline declined from the first year to the third (21.21%, 12.95% and 9.58%, respectively).

The equal representation of each patriline in the worker progeny was consistently rejected for every year as well as for the overall sample of all 982 workers (50.42 < χ^2^ < 336.76, 16 df, *p* < 0.001, Chi^2^ test). This result is either due to unequal number of sperm of drones stored in the spermatheca, or if contributions of single drones are expected to be approximately equal, the consequence of processes taking place after mating.

As an index that is unaffected by sample size, the variance of the relative frequency of each patriline was calculated for all three years. In our experimental colony the variance declined from the first to the third year: 0.0025, 0.0015 and 0.0009 for 1998, 1999 and 2000 respectively (Table 1), which shows that patrilines were more equally represented in the third than in the second or first year.

The number of effective matings (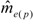) was calculated according to Pamilo [46] 


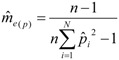
(1)

where *n* is the number of sampled worker bees, and *p_i_* is the estimated relative proportion of offspring sired by drone *i*. The effective paternity frequency increased from the first to the third year: from 10.64 to 12.29 and 14.27, respectively. The estimated number of matings is always less than the actual number of patrilines detected, unless all patrilines are equally represented. The error of 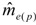
decreases as sample size *n* increases. The ratio of 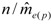
can be used as a standard measure for the magnitude of the error. Tarpy and Nielsen [22] defined a threshold at which the upper 95% confidence level is £ one effective drone father. The ratio of 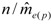
in the three investigated years is 15.51, 45.26 and 18.29, respectively, and always exceeds the threshold with the particular 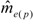
(Table 1), calculated from the logarithmic function: 


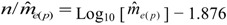
(2)

The 95% range of 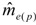
can be calculated from [22]: 


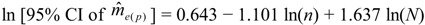
(3)

where *n* is the number of sampled workers and *N* is the effective paternity frequency. 

While the estimated effective number of matings increased, the average coefficient of genetic relatedness, which is not independent of 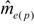
decreased from the first to the third year: intracolonial relatedness between workers *Ĝ* was calculated from Laidlaw and Page [47] using


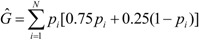
(4)

where *p_i_* is the frequency of the *i*th patriline. Intracolonial genetic relatedness was 0.300, 0.292 and 0.285, in the first, second and third year, respectively.

**Table 1:** Absolute frequency of patrilines (number of sampled workers belonging to each patriline) among the offspring of a single queen followed for three years, number of workers sampled, variance of relative frequencies of patriline distribution, number of effective matings [46], ratio 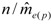, threshold and 95% CI calculated according to [22], and intracolonial relationship [47].

	Year		
	1998	1999	2000
Patriline A	35	71	22
Patriline B	21	59	20
Patriline C	14	50	18
Patriline D	14	72	24
Patriline E	12	26	15
Patriline F	10	36	17
Patriline G	10	39	25
Patriline H	9	18	19
Patriline I	8	25	19
Patriline J	7	26	9
Patriline K	7	35	19
Patriline L	5	20	8
Patriline M	4	49	23
Patriline N	3	14	18
Patriline O	3	8	3
Patriline P	2	0	0
Patriline Q	1	8	2
Number of workers	165	556	261
Variance	0.0025	0.0015	0.0009
Effective matings 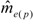	10.64	12.29	14.27
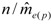	15.51	45.26	18.29
Threshold	7.45	8.02	8.61
95% CI	0.52	0.19	0.53
Intracolonial relationship	0.300	0.292	0.285

## 4. Discussion

If sperm usage by honey bee queens is random, as indicated by previous researchers, the subfamily ratios in a honey bee colony should theoretically remain constant over time. Authors using molecular methods to analyze the patrilines of a honey bee colony failed to find evidence of sperm clumping or any form of significant changes in sperm usage. Page and Metcalf [1] studied sperm usage weekly over a period of 11 weeks, and detected no temporal changes. This coincides with our results, although they used an allozyme method, allowing them to assign a queen’s progeny to 3 distinguishable phenotypes only. Estoup *et al*. [18] were the first to use microsatellite markers in the honey bee, but also failed to demonstrate any long-term changes in patriline distribution, possibly because low sample numbers prevented the detection of differences. Haberl and Tautz [32] could not find any evidence for sperm clumping by analyzing sequentially laid eggs. After repeating their experiment 12 months later using the same honey bee queen, they found differences between the samples, which they attributed either to different sampling techniques (pupae and alternatively eggs and pupae) or to shifts in sperm use over years.

Our results confirm the findings that there is no change in short-term sperm utilization, as shown on the fine scale by Haberl and Tautz [32] and for the first months of a queen’s life by Franck *et al*. [39]. In contrast to previous authors, who used young queens, we analyzed for the first time the patrilines of a honey bee colony at the queen’s age of two, three and four years. Although we only investigated one colony, our results are the first to reveal significant long-term differences in the patriline distribution of a honey bee colony. 

In our experiment the number of effective matings 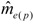
increased with time, and the variance of patriline ratios decreased with time. The latter finding is supported by the data of Franck *et al*. [39], who concluded that the patriline composition “evolves in a progressive way” in the first three months of a queen’s life. Franck *et al*. [48] also found decreasing variance for some colonies over a period of twelve months, which they attributed to the spermatozoa of different drones not being completely mixed. Intracolonial relatedness between workers, which depends on the number of matings, decreased with time. When we recalculated the data of Franck *et al*. [39] we found the same tendency. These trends, especially the decrease of variance, are due to a more equal representation of patrilines with time, which could be caused by mixing processes in the queen’s spermatheca.

Kerr *et al*. [31], who used two distinct worker genotypes (Italian and *carnica*), demonstrated temporal genetic fluctuations of those genotypes in a multitude of colonies. Referring to Kerr’s data, Page *et al*. [49] pointed out that fluctuations between genotypes decreased over time, a result which agrees with our data of decreasing variances of patriline ratios. One conclusion of our data is that infrequent patrilines increase with time, whereas very frequent patrilines become less dominant from one year to the next. Rare patrilines, close to the limit of detection, didn't change their proportional representation significantly. From this we conclude that either some drones contribute less to the total amount of sperm a queen receives during her nuptial flights, or progeny of these drones are underrepresented through other mechanisms following copulation. Former investigations using molecular methods, as well as our experiment, reject the sperm-clumping hypothesis because multiple patrilines are present at any time.

The tendency toward equalization of some patrilines over longer periods is conceivable through one or more of the following mechanisms: (1) Adaptions to sperm competition. In the honey bee, ejaculate competition has been demonstrated for the first days after mating in the Bursa copulatrix and the oviducts before the majority of the sperm is expelled and only a small portion is stored in the spermatheca [49]. On the other hand, spermathecal fluids maximize the survival of all sperm [50], hence sperm competition inside the spermatheca is improbable and there is also no empirical proof [51]. (2) Queen control. We do not want to rule out the possibility that the honey bee queen has control over which drone’s spermatozoa are chosen for insemination but nothing is known about a mechanism existing for this kind of anatomical or physiological process. (3) Sperm mixing processes in the queen’s spermatheca. Among others, Tarpy and Nielsen [22] point out that differential fertilisation may also be the result of incomplete mixing of sperm. For example, sperm stored in a spermatheca for two years show slower and different movement than fresh sperm and spermathecal enzymatic activities change over time [52] as well as there is an increase in the percentage of non-viable sperm [53].

Taber [42] took it for granted that “sperm of different drones does not mix appreciably” and early studies demonstrated the incomplete mixing of sperm [31,42] but no clumps, layers, aggregations or wads could be visually detected in the queen’s spermatheca [54]. We also agree with and extend the hypothesis of sperm dispersal and filling of the spermatheca as described by Page *et al*. [54] and Page [12]: After the queen returns to the hive from her mating flights, the spermatozoa migrate from the lateral and median oviducts towards the spermatheca. It is likely that the first spermatozoa reaching the spermatheca have plenty of room to disperse, but as the spermatheca gets more densely packed, there is less room for the spermatozoa to disperse. Spermatozoa deposited at the anterior ends of the lateral oviducts are the last to reach the spermatheca and have less room to distribute. As spermatozoa become more densely packed, the probability of sperm of the same drone to be closely associated with each other increases. About 40 h after the queen’s mating, the filling of the spermatheca is finished [27], and at this time sperm may not be completely mixed.

Every time the queen lays an egg, a small volume of fluid containing spermatozoa is released from the spermatheca. Harbo [30] stated that the volume released for each egg is constant, namely 1/153,000 of the total spermathecal volume (about 5,000 µm^3^). As a consequence the number of spermatozoa remaining in the spermatheca declines in a non-linear way, expressed by the equation *ln a_t_ = ln a_0_ − t (1/153,000)*, where *a_t_* is the number of spermatozoa in the spermatheca after a queen has laid any number *(t)* of eggs and *a_0_* is the original number of sperm in the spermatheca after mating.

The volume of the spermatheca is kept constant by replacing the released volume by fluid [30]. This may promote further mixture of spermatozoa still remaining in the spermatheca in two ways: (1) the packing density inside the spermatheca drops and spermatozoa have more room to disperse, and (2) the replacement of spermatozoa and fluid with fluid only reduces viscosity of the spermathecal content.

The age of a honey bee queen affects the total accumulated number of laid eggs, and thereby the dispersion of spermatozoa of different drones within the spermatheca. As we have not measured the number of eggs laid, we want to estimate this number, to give an example of how drastically packing density of spermatozoa drops with time. We assume that a honey bee queen has stored the characteristic number of about 5 million spermatozoa after matings and in the course of two years lays about 300,000 eggs [23]. Applying Harbo’s equation [30], about 4.3 million spermatozoa will thereby be released for fertilization within two years, with only about 700,000 spermatozoa still remaining in the spermatheca. After the production of another 150,000 eggs, which roughly is the number laid in one year, the spermatheca contains only about 260,000 spermatozoa. Page [12] argues that a honey bee queen may produce about 200,000 fertile eggs that develop into sterile workers before she lays “the few eggs that eventually develop into queens”. Accordingly we conclude that dispersed spermatozoa gain more fitness, because there will be a higher probability for them to fertilise an egg laid into queen cells in reproduction periods.

Extending Harbo’s [30] hypothesis of non-linear decrease of spermatozoa, we conclude that the number of spermatozoa released to fertilize eggs in the early stage of a queen’s life is higher, compared to later in her life when she has already laid a multitude of eggs. We calculated the average number of spermatozoa to be released per egg by dividing the total number of released spermatozoa by the number of fertilized eggs, which can be expressed as


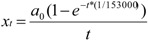
(5)

where *x_t_* is the average number of spermatozoa released for fertilization of egg number *t*. Again, we assume that a queen has about 5 million spermatozoa in the spermatheca. The average number of spermatozoa included in the (constant) volume released to fertilize one egg drops from about 32 for one of the first few eggs laid, to about 10 after laying about 500,000 eggs. Maybe the broad range of early reports of the number of sperm released to fertilize one egg in the honey bee (see references in [30,55]) is due to this decline. In leaf-cutter ants, a decrease of the number of sperm used per egg has only been demonstrated for young founding queens compared to established queens whereas, in contrast to our assumption, median sperm use increased with age of established queens [55]. We speculate that the volume of spermatozoa and fluid released from the spermatheca for fertilisation may be an arena for sperm competition. As the number of spermatozoa drops with the total accumulated number of eggs laid, the probability of spermatozoa of a given drone being included in the released volume also decreases (although the probability of outcompeting the other sperm in the released volume to fertilize an egg increases).

## 5. Conclusions

In contrast to earlier studies demonstrating fluctuations in patriline distribution [31,42], our study was more extensive, used microsatellite markers, and included a queen up to the age of four years. We conclude that, in some disagreement with previous investigations of single colonies using molecular markers [18,32,39], the patriline distribution of a honey bee colony may change over long periods of time. Hence further research efforts, for example regarding the efficiency of sperm use for fertilization of eggs by young and old honey bee queens [30,55], are needed to help understand a topic that has been investigated for decades.
